# Severe fever with thrombocytopenia syndrome: a systematic review and meta-analysis of epidemiology, clinical signs, routine laboratory diagnosis, risk factors, and outcomes

**DOI:** 10.1186/s12879-020-05303-0

**Published:** 2020-08-05

**Authors:** Zhiquan He, Bohao Wang, Yi Li, Yanhua Du, Hongxia Ma, Xingle Li, Wanshen Guo, Bianli Xu, Xueyong Huang

**Affiliations:** 1 College of Public Health, Zhengzhou University, Zhengzhou, China; 2 Henan Province Center for Disease Control and Prevention, Zhengzhou, China; 3 Henan Key Laboratory of Pathogenic Microorganisms, Zhengzhou, China

**Keywords:** SFTS, Clinical signs, Routine laboratory diagnosis, Risk factors, Epidemiology, Outcomes, Meta-analysis

## Abstract

**Background:**

Severe fever with thrombocytopenia syndrome (SFTS) is an emerging infectious disease with the high case-fatality rate, and lack of vaccines. We aimed to systematically analysed the epidemiological characteristics, clinical signs, routine laboratory diagnosis, risk factors, and outcomes.

**Methods:**

Documents on SFTS were collected by searching the Chinese National Knowledge Infrastructure, Wan Fang Data, PubMed, Embase, and Web of Science databases from 2011 to 2018. Meta-analysis was performed by using Review Manager and Stata software.

**Results:**

Twenty-five articles involving 4143 cases were included. Diarrhea (odds ratio (OR) =1.60, 95% confidence interval (CI): 1.06 to 2.42, *P* = 0.02), and vomiting (OR = 1.56, 95% CI: 1.01 to 2.39, *P* = 0.04) on admission were associated with the fatal outcomes of SFTS. Compared to patients with mild symptoms, patients with severe symptoms had significantly elevated levels of lactic acid dehydrogenase (standard mean difference (SMD) =1.27, 95% CI: 0.59 to 1.94), alanine aminotransferase (SMD = 0.55, 95% CI: 0.24 to 0.85), aspirate aminotransferase (SMD = 1.01, 95% CI: 0.69 to 1.32), and creatine kinase (SMD = 1.04, 95% CI: 0.74 to 1.33) but had reduced platelet counts (SMD = -0.87, 95% CI: − 1.16 to − 0.58) and albumin levels (SMD = -1.00, 95% CI: − 1.32 to − 0.68). The risk factors for poor prognosis included age (mean difference (MD) =6.88, 95% CI: 5.41 to 8.35) and farming (OR = 2.01, 95% CI: 1.06 to 3.80). For the risk factors of contracting SFTS, the incidence of SFTS related to tick bites was 24% [95% CI: 0.18 to 0.31]. The pooled case-fatality rate of SFTS patients was 18% [95% CI: 0.16 to 0.21].

**Conclusions:**

China is the country with the highest incidence of SFTS. May to July was the peak of the epidemic, and farmers were a high-risk group. The risk factor for SFTS included age (poor prognosis) and tick bites (contracting SFTS). Patients with severe diarrhea and vomiting symptoms on admission should be noted. Clinicians could use routine laboratory parameters and clinical symptoms as references for clinically suspected cases, classification of SFTS, and timely treatment, especially in basic hospitals.

## Background

Severe fever with thrombocytopenia syndrome (SFTS) is a novel emerging infectious disease that was first discovered in rural areas of eastern and central China. In 2009, a novel bunyavirus was isolated from acute-phase patient serum samples and named as severe fever with thrombocytopenia syndrome virus (SFTSV), huaiyangshan virus (HYSV), or new bunyavirus (NBV) [1–4]. It is now known as SFTSV. The disease has been reported in 23 Chinese provinces, more than 5000 cases were reported during 2009–2016, and Henan province had the highest case count, accounting for 45% of reported cases during 2011–2014 in China [2, 5, 6]. SFTS patients have been found in Japan and Korea [7, 8]. SFTSV is transmitted by tick bites, and human-to-human transmission has also been reported [9–12]. A high case-fatality rate, ranging from 5 to 20%, has been reported for SFTSV-infected patients in the endemic areas [13–15].

SFTS has been a serious public health concern; however, effective therapies or vaccines are not yet available, so understanding its features has important significance for the prevention and treatment of disease. In this article, we systemically searched and analysed the epidemiology, clinical signs, routine laboratory diagnosis, risk factors, and outcomes of SFTS.

## Methods

### Literature searching

We carefully performed a systematic search of the Chinese National Knowledge Infrastructure databases (CNKI), Wan Fang Data, PubMed, Embase, and Web of Science databases for all eligible papers (published from 2011 to 2018; English and Chinese publications) using the following search terms: “severe fever with thrombocytopenia syndrome” OR “SFTS” OR “SFTSV” OR “NBV”. We also manually included additional studies obtained from the references of the original articles and searches.

### Inclusion and exclusion criteria

An initial screening of the titles and abstracts was performed by two authors independently. Thereafter, two independent authors screened the full texts of the selected articles. The inclusion criteria were as follows: the article had been accepted for publication; the study provided information on SFTS patients, or the SFTS patient mentioned in the selected studies was confirmed as meeting one or more of the following criteria: (1) the virus was isolated from the patient’s samples; (2) SFTSV RNA was detected in the patient’s serum; (3) a 4-fold or greater increase in antibody titers was detected between a paired patient serum samples collected from the acute and convalescent phases of infection.

The exclusion criteria included abstract-only articles, case reports (*n* < 5), letters, editorials, systematic reviews, duplicated publications, overlapping data sets (articles of the same region and year or articles containing another article were collected and carefully selected by two authors), in vitro studies, studies on animals, genotype analyses or treatments, and articles in which no data were extracted.

### Data extraction and quality assessment

Data were independently extracted by two reviewers from the included articles. Disagreement was resolved through discussion or consensus. Subsequently, the following information was extracted from every eligible article: the first author; year of publication; region; year of admitted patients; the timing of the symptoms; number of patients; patient’s age; clinical information about SFTS patients, including symptoms (fever, myalgia, sputum, anorexia, abdominal pain, diarrhea vomiting, fatigue, gingival bleeding, headache, cough, nausea, petechiae and lymphadenopathy), routine laboratory parameters (lactic acid dehydrogenase (LDH), alanine aminotransferase (ALT), aspirate aminotransferase (AST), platelet count, albumin, creatine kinase (CK), creatinine, and white blood cell (WBC) count); interval between onset and admission; risk factors; outcomes; and the number of deaths. In addition, if there was no reliable data, we input “NA”, which means “not available”, during the extraction phases.

We evaluated the quality of primary studies using Study Quality Assessment Tools provided by the National Institute of Health [16]. The criteria were divided into three grades: good, fair and poor. Based on the quality assessment for studies, we identified the articles’ quality ratings.

### Statistical analysis

Means and standard deviation (SDs) were chosen to describe continuous variables with normal distributions. The medians and interquartile (IQ) ranges are shown for the interval between onset and admission, laboratory parameters and age. If the data showed medians and ranges or IQ ranges rather than the means and SDs, then the means and SDs were calculated as described by Hozo et al., Wan et al., and Luo et al. [17–19]. Each study presenting the number of patients was included for calculation of the event rates and proportions with 95% confidence intervals (CIs) for clinical signs and outcomes. I-squared and Chi-square were chosen to reflect the heterogeneity among these studies [20]. Heterogeneity was considered significant when *P* < 0.05 and I^2^ > 50%; then, a random effect model was applied. Otherwise, a fixed effect model was used. Furthermore, publication bias was assessed by visually using Begg’s funnel plot or Egger’s test [21]. All of the statistical analyses were performed using Review Manager (RevMan version 5.3; Nordic Cochrane Centre, Copenhagen, Denmark) software and STATA software version 12.0 (STATA Corporation, College Station, Texas, USA).

## Results

### Systematic review

**Fig. 1:**
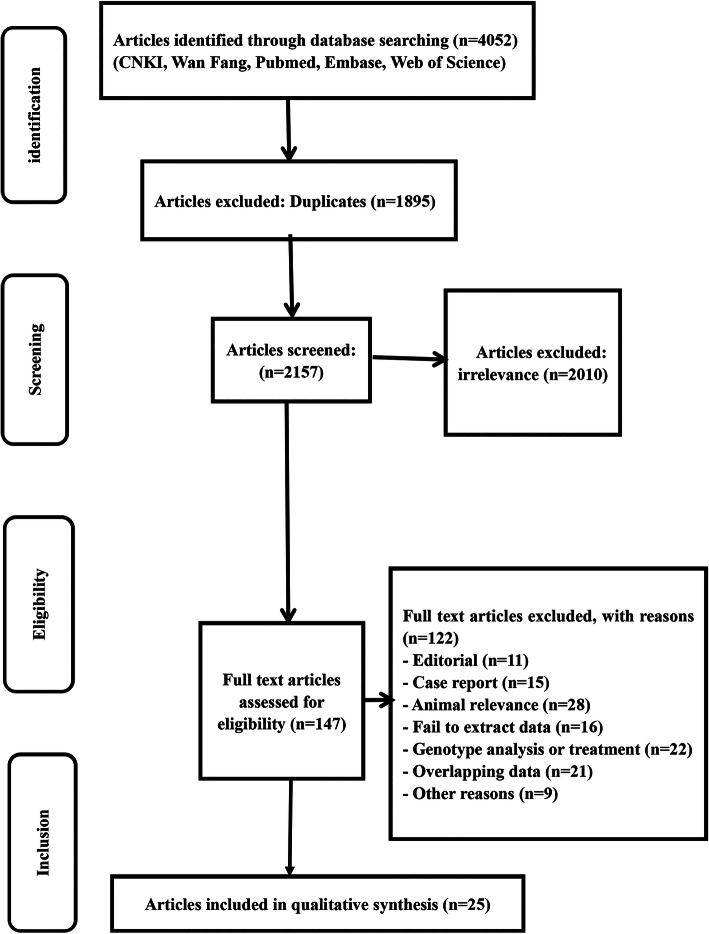
A flow diagram showing the selection of studies

The study selection process and the results are shown in Fig. 1. A total of 4052 articles were retrieved after the preliminary screening from the electronic databases; 1895 articles were duplicated and removed, and 2010 of the 2157 articles were excluded after review of the titles and abstracts due to irrelevant topics (the sinonasal solitary fibrous tumours). After reading the full texts of the remaining 147 articles, 122 articles were excluded due to lack of some indicators. Finally, 25 studies were included for further meta-analysis. The detailed data are shown in Table 1. In this review, the largest numbers of reported cases and articles were from China (21 articles, 3876 cases), followed by Korea (3 articles, 218 cases) and Japan (1 article, 49 cases). In a period of 8 years, from 2011 to 2018, 21 articles in China were collected, which included Henan province (2 articles), Jiangsu province (4 article), Liaoning province (2 article), Shandong province (3 article), Hubei province (5 articles), Zhejiang province (2 article), Anhui province (2 article), and undetermined province (1 article). The remaining 4 articles were Korea (3 articles) and Japan (1 article). For the season, 10 of 25 articles suggested that the epidemic peak was in May to July. In terms of geographical and time trends, the two points were considered important sources of potential bias when explaining the evidence for SFTS.

**Table 1:** Characteristics of included studies in Meta-analysis

Study	Publication year	Region	Year of admitted patients	Age	Case Number	Death Number	Quality Rating	interval between onset and admission
Bao et al. [22]	2011	Jiangsu, China	2007	61.4 ± 10.5^a^	7	1	Poor	4 (3–7.5)^b^
Deng et al. [23]	2012	Liaoning, China	2011	55.7 ± 14.7	40	6	Poor	5.5 (1–10)^d^
Gai et al. [24]	2012	Shandong, China	2008–2011	61(40–83)^d^	59	11	Fair	NA
Zhang et al. [25]	2012	Hubei, China	2010	54.4 ± 9.1	49	8	Fair	10 (7–14)^b^
Deng et al. [26]	2013	Liaoning, China	2010–2011	55 (17–89)^d^	115	14	Good	5 (1–14)^d^
Sun et al. [27]	2013	Hubei, China	2012	55(30–78)^d^	34	3	Fair	NA
Ding et al. [28]	2014	NA	2010–2011	61 (23–82)^d^	59	7	Fair	6 (1–13)^d^
Sun et al. [29]	2014	Zhejiang, China	2011–2013	66(31–84)^d^	65	9	Fair	NA
Xu et al. [30]	2015	Henan, China	2007–2011	51.4 ± 13.0	422	68	Fair	NA
Shin et al. [31]	2015	South Korea	2013	69 (28–84)^d^	35	16	Fair	4 (1–9)^d^
Choi et al. [32]	2016	South Korea	2013–2015	67.5 (57–76)^b^	172	56	Good	4 (3–6)^b^
Kato et al. [33]	2016	Japan	2013–2014	78 (65–84)^b^	49	15	Good	4 (2–5)^b^
Peng et al. [34]	2016	Hubei, China	2014	53.8(24–79)^d^	53	9	Poor	NA
Xiong et al. [35]	2016	Hubei, China	2015	58(27–91)^d^	179	34	Good	NA
Xu et al. [36]	2016	Shandong, China	2011–2015	62.1 ± 10.6	113	21	Fair	NA
Zhao et al. [37]	2016	Jiangsu, China	2010–2014	57.6(38–78)^d^	40	7	Fair	NA
Zhang et al. [38]	2016	Hubei, China	2015	60(28–91)^d^	115	21	Poor	NA
Hu et al. [39]	2017	Jiangsu, China	2011–2013	56.5(76–83)^d^	89	19	Fair	8.5 (1–50)^b^
Hu et al. [40]	2018	Zhejiang, China	2014–2017	57.8 ± 12.662	25	5	Fair	6 (0–11)^b^
Jia et al. [41]	2018	Jiangsu, China	2010–2016	59 (51–67)^b^	90	20	Fair	8 (7–9)^b^
Song et al. [42]	2018	Anhui, China	2011–2017	64(24–86)^d^	87	12	Poor	NA
Xia et al. [43]	2018	Anhui, China	2014–2017	62 ± 10.82	86	12	Fair	NA
Li et al. [44]	2018	Henan, China	2011–2017	61.4 ± 12.2	2096	340	Good	5 (4–7)^b^
Kwon et al. [45]	2018	Korea	2015–2016	60 ± 7	11	1	Fair	NA
Xu et al. [46]	2018	Shandong, China	2014–2015	65.82 ± 11.36	60	20	Good	6 (4–7)^b^

^a^values are listed as Mean ± SD (standard deviation), ^b^values are listed as Median (IQ), ^c^means not available, ^d^values the Median (Range)

### Clinical symptoms of SFTS

**Fig. 2:**
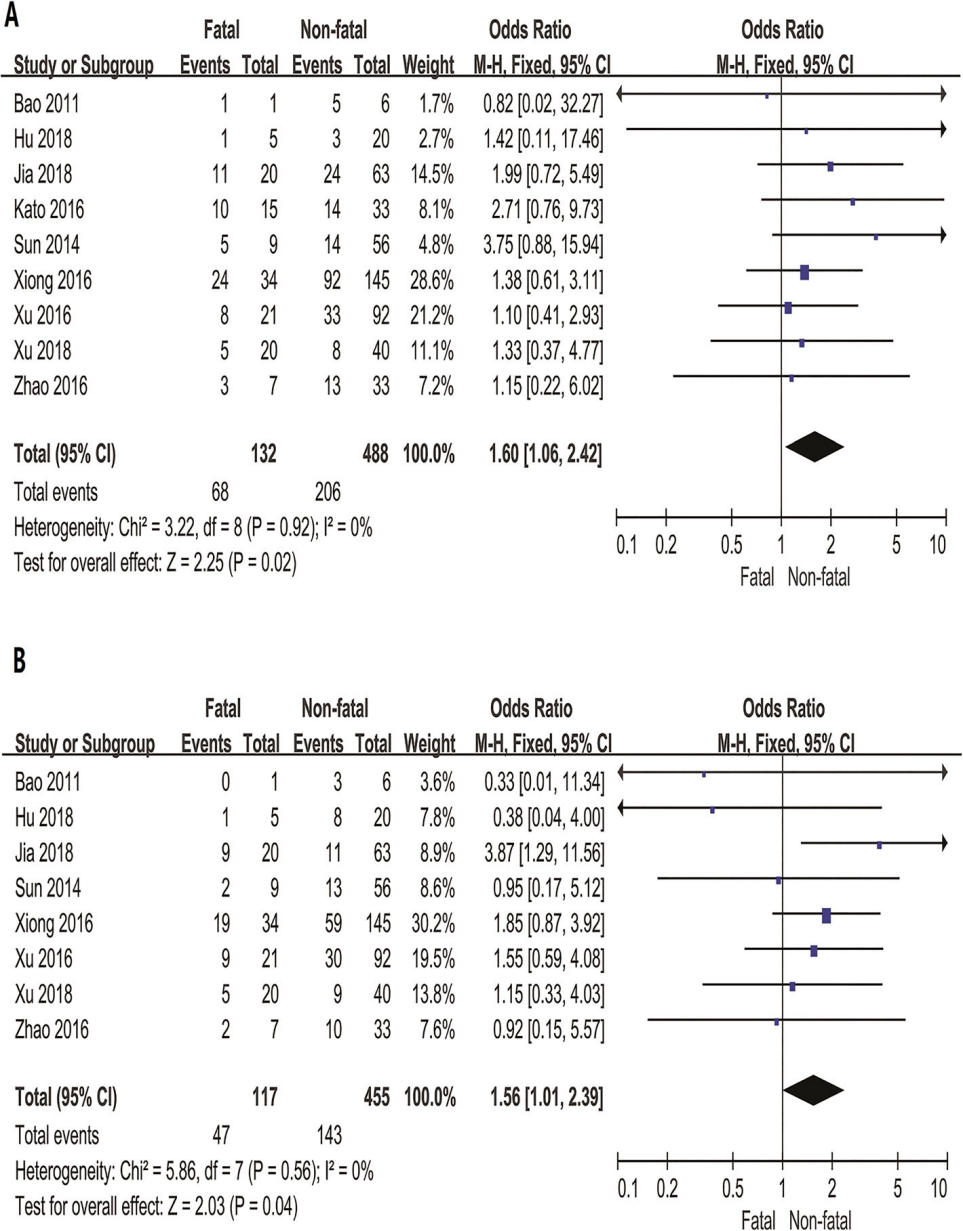
Forest plots for the meta-analysis of a panel of the clinical signs and fatal outcomes of SFTS disease. **a** Diarrhea (on admission), **b** Vomiting (on admission)

The clinical symptoms were more representative in the comparison of fatal and non-fatal SFTS patients: 12 articles, including 887 cases, presented the clinical characteristics and were analysed and compared. Based on the timing of the symptoms, the studies were divided into two groups focusing either on admission (9 studies) or during the hospitalization (3 studies). Since the definition of fever was different in various studies, so we did not consider fever symptoms. Diarrhea and vomiting on admission were associated with the fatal outcomes of SFTS disease (OR = 1.60, 95% CI: 1.06 to 2.42, *P* = 0.02; OR = 1.56, 95% CI: 1.01 to 2.39, *P* = 0.04), respectively (Fig. 2). However, there was no significant heterogeneity in other signs either on admission or during hospitalization (Table S1).

Fourteen pooled positive rates of clinical symptoms were calculated, and 15 studies focusing on admission used the random effect model due to symptoms, with I-squared values > 50%. The included studies, pooled positive rates (95% CI), *P* values (I-squared) and sensitive analyses are presented in Table S2.

### Routine laboratory diagnosis of SFTS

**Fig. 3:**
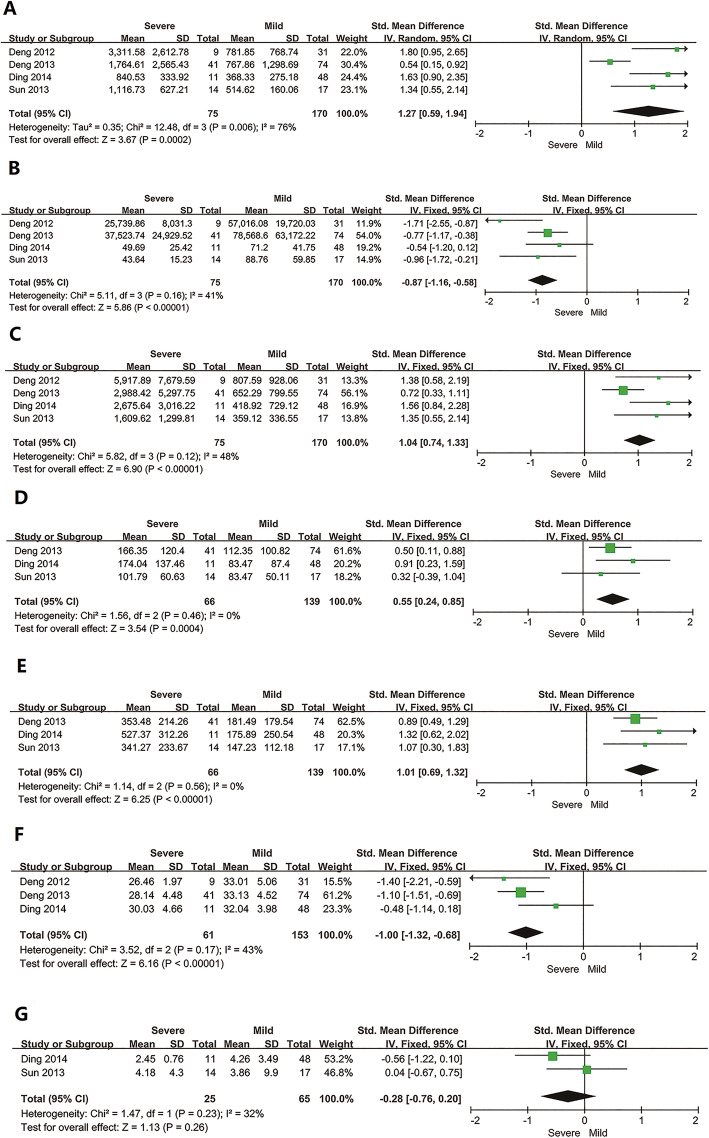
Forest plots of meta-analysis on a panel of routine laboratory parameters. **a** LDH, **b** Platelet count, **c** CK, **d** ALT, **e** AST, **f** Albumin, **g** WBC count

Mild cases were accompanied with fever (37.2 °C–39 °C), fatigue, gastrointestinal symptoms, leukocyte count decreased, platelet count was (50–130) × 10^9^/L, and the levels of AST, ALT, CK, and LDH were less than two times the upper limit of normal (ULN) values. Severe cases had the high fever (39 °C–40 °C), fatigue, obvious gastrointestinal symptoms, neurological symptoms, platelet count was (30–50) × 10^9^/L, and sharply elevated (more than five times ULN) of LDH, ALT, AST, and CK. As shown in Fig. 3, the routine laboratory findings of patients with severe and mild symptoms were compared. Patients with severe symptoms had significantly elevated levels of LDH (SMD =1.27, 95% CI: 0.59 to 1.94), ALT (SMD = 0.55, 95% CI: 0.24 to 0.85), AST (SMD = 1.01, 95% CI: 0.69 to 1.32), and CK (SMD = 1.04, 95% CI: 0.74 to 1.33) compared to the patients with mild symptoms but showed reduced levels of platelets (SMD = -0.87, 95% CI: − 1.16 to − 0.58) and albumin (SMD = -1.00, 95% CI: − 1.32 to − 0.68). There were no differences in mild and severe SFTS cases regarding the WBC count (SMD = -0.28, 95% CI: − 0.76 to 0.2). The one study showed that serum creatinine in 115 patients’ blood ranged from 4.9 to 370 μmol/L. The median creatinine was 74.9 μmol/L in 74 patients with mild symptoms, and 93 μmol/L in 41 patients with severe symptoms. The serum creatinine was significantly higher in patients with severe symptoms compared with the patients with mild symptoms (*P* < 0.05) [26].

### Risk factors for SFTS

**Fig. 4:**
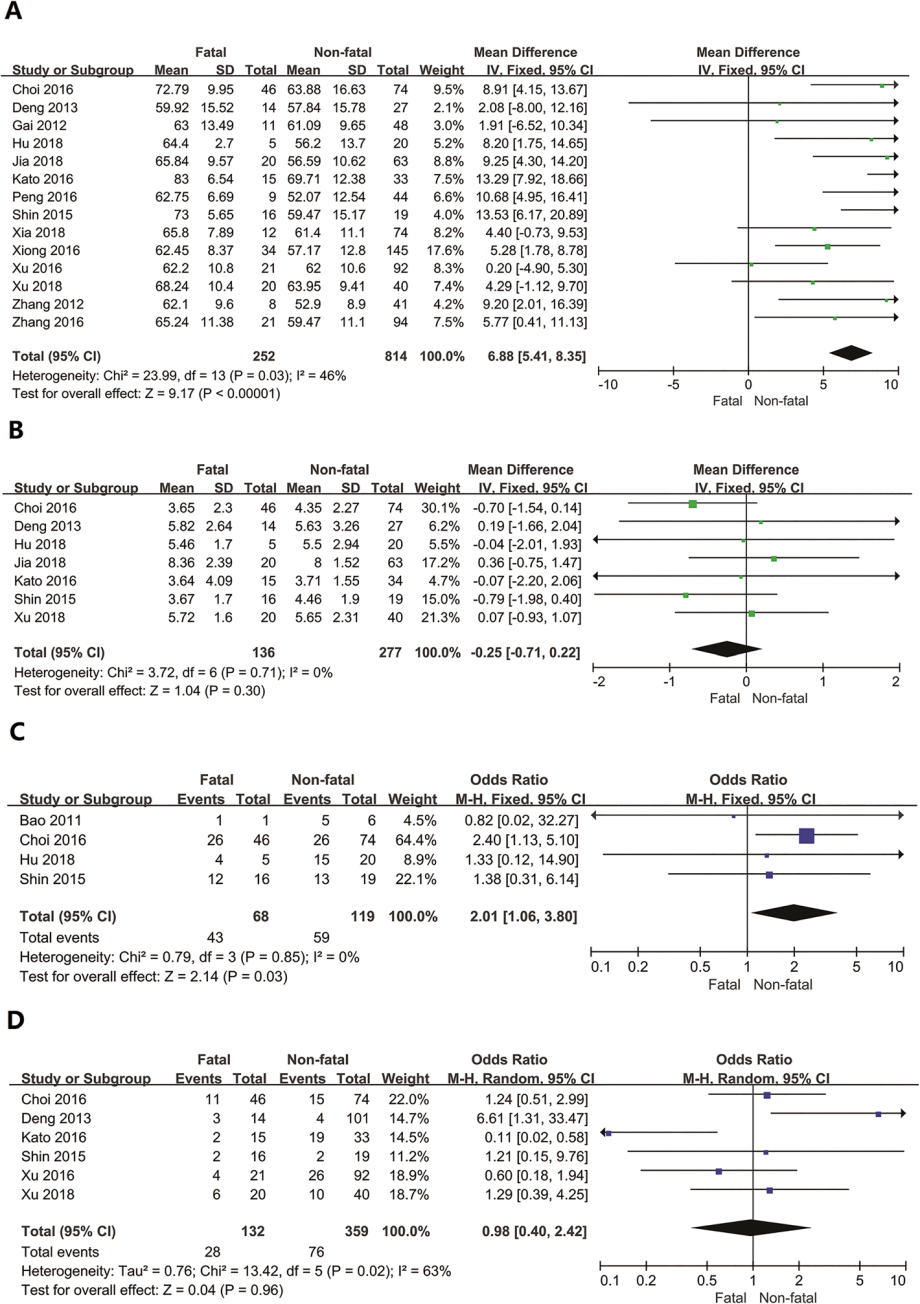
Forest plots of meta-analysis on a panel of risk factors. **a** Age, **b** Interval between onset and admission, **c** Farming, **d** Tick bite

For the risk factors of poor prognosis, we analysed age, the interval between onset and admission, farming and tick bites. Age was the critical risk factor for SFTS patients (MD =6.88, 95% CI: 5.41 to 8.35), but the interval between onset and admission had no significant association with the disease (MD = -0.25, 95% CI: − 0.71 to 0.22). Engaging in agricultural activity was a risk factor, resulting in the occurrence of death case (OR = 2.01, 95% CI: 1.06 to 3.80). One hundred thirty two fatal cases and 359 non-fatal cases were extracted to study the relationship between tick bites and fatal outcomes. The results showed that tick bites were not a risk factor for death case (OR = 0.98, 95% CI: 0.40 to 2.42), and the fatality due to severity of the disease and personal conditions. For the SFTS cases, tick bites play a key role in the risk of infection, which were considered to be the main route of transmission of SFTSV. The 11 articles were analysed and showed that 24 % [95% CI: 0.18 to 0.31] (284 biters in 1228 cases) had been bitten by ticks, indicating the incidence of SFTS related to tick bites (Fig. 4).

### Outcomes of SFTS patients

**Fig. 5:**
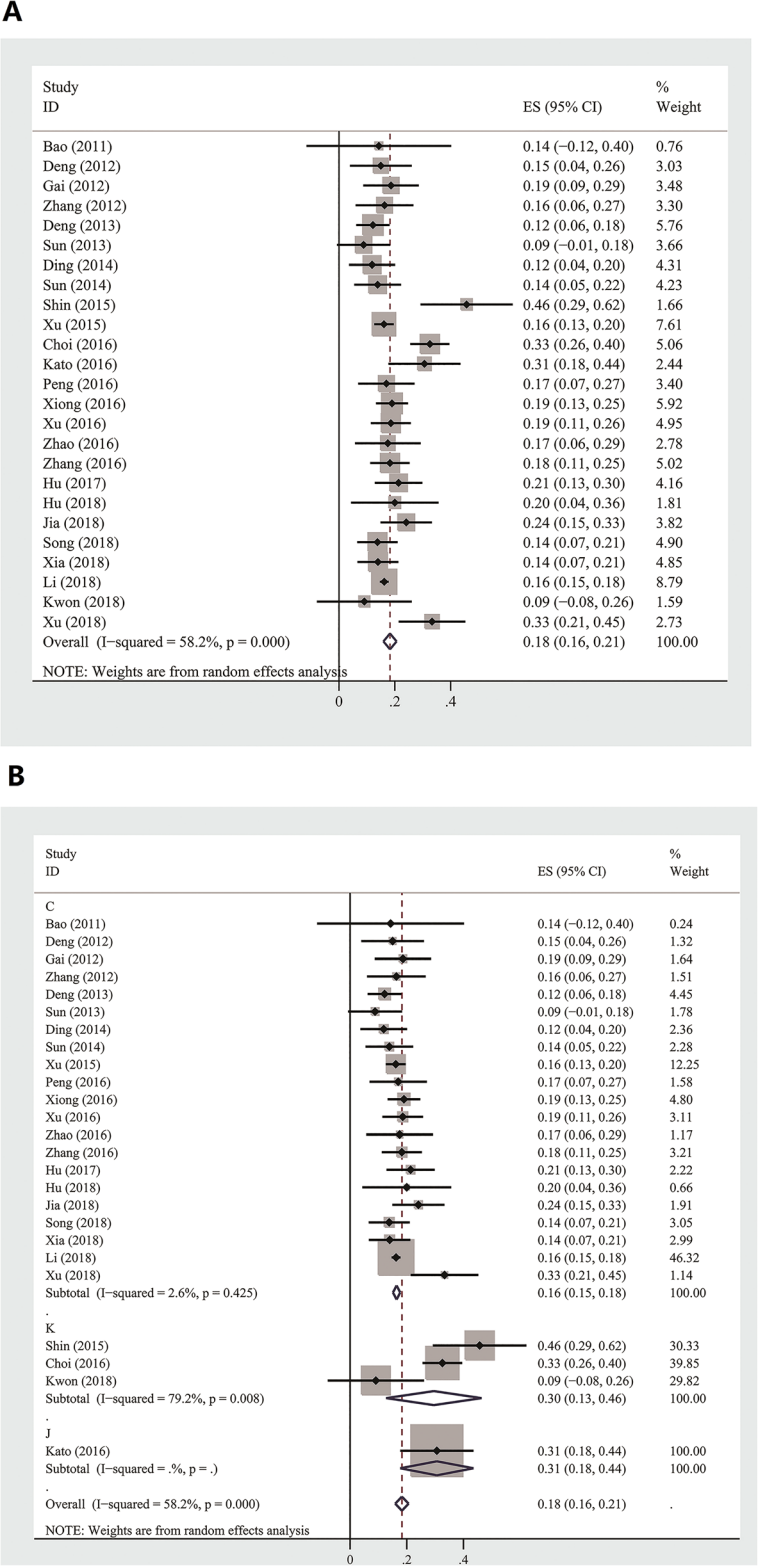
**a** Forest plot of case fatality rate for SFTS patients, **b** Subgroup analysis forest plot according to location

The pooled case-fatality rate of SFTS patients was 18% [95% CI: 0.16 to 0.21] (735 deaths in total among 4143 cases). According to country (China, Japan, and Korea), the pooled mean mortality rates were 0.16 [95% CI: 0.15 to 0.18], 0.31 [95% CI: 0.18 to 0.44], and 0.30 [95% CI: 0.13 to 0.46], respectively. In the heterogeneity analysis, the I-squared value was 58.2%, suggesting that there was significant heterogeneity among these studies, and a random effects model was adopted (Fig. 5a). Subsequently, subgroup analysis was applied based on the location (China, Korea, Japan), and there was no heterogeneity in China (I^2^ = 2.6%, *P* = 0.425); However, articles from Korea had significant heterogeneity (I^2^ = 79.2%, *P* = 0.008) (Fig. 5b).

### Sensitivity analysis and publication bias

Clinical signs were pooled for positive rates on admission, and sensitivity analysis was performed, which indicated that the data changed little (Table S2). The case fatality rate in the sensitivity analysis forest plot was stable and had no significant effect on the merger rate (the combined rate was 18, 95% CI: 0.16 to 0.21).

**Fig. 6:**
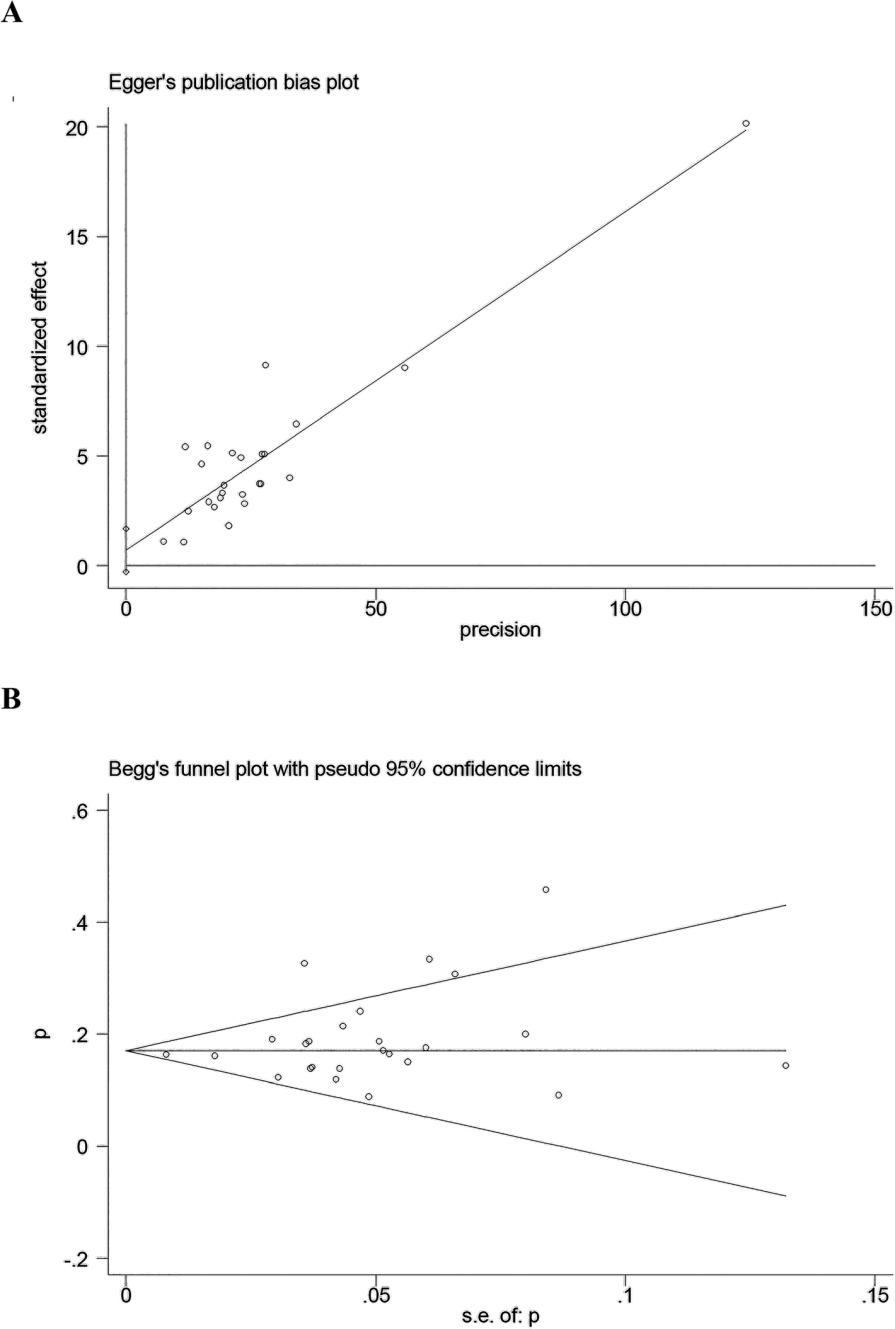
Funnel plots for publication bias. **a** Egger’s publication bias plot, **b** Begg’s funnel plot

Egger’s test and Begg’s test were conducted to evaluate the publication bias, including 25 articles. The results showed that the Egger’s test t value was 1.50 (*P* = 0.147), and the Begg’s test z value was 1.14 (*P* = 0.252) (Fig. 6). Because the proportion of the fortieth reference datum was relatively high, when we removed it, the results showed the Egger’s test t value was 1.22 (*P* = 0.235), and the Begg’s test z value was 1.17 (*P* = 0.244).

## Discussion

Our review identified a total of 4143 SFTS cases from 25 articles in SFTS- endemic regions including China, Korea and Japan. The main clinical and laboratory characteristics of SFTS were fever, gastrointestinal and neurological symptoms as well as thrombocytopenia and leukopenia. Fever was the most common symptom of SFTS patients, suggesting that it is an important indicator of the early stage of disease. The gastrointestinal and neurological symptoms included anorexia, nausea, vomiting, headache and other signs, but these symptoms were not very specific and were not associated with the progression of the disease; Further, some viral hemorrhagic fevers could cause these common symptoms, such as Rift Valley, Dengue fever and haemorrhagic fever with renal syndrome (HFRS) [47–49]. The clinical signs of fatal and non-fatal SFTS patients were studied, and the results showed that diarrhea and vomiting on admission were associated with fatal outcomes. Diarrhea and vomiting were obvious symptoms for patients, and most patients had these clinical symptoms. Clinicians could take appropriate treatment measures quickly on admission. The clinical signs, such as diarrhea and vomiting, had no differences during hospitalization. The reason might be that patients with clinical symptoms would be treated in time during hospitalization, while most of the patients had clinical symptoms before admission, so the treatment was delayed and the condition aggravated. In the intensive care unit (ICU) or ward, clinicians should pay special attention to patients with diarrhea and vomiting and timely treatment to prevent the disease from aggravating. We extracted the numbers of positive symptoms and total cases on admission to calculate the pooled positive rates of clinical symptoms. Meta-analysis showed that most clinical signs had significant heterogeneity, and the random effect model was used. Subgroup analysis explored the source of heterogeneity, and the results suggested that different provinces had impacts on heterogeneity. China has a vast territory and the incidence of SFTS was widespread. Although we issued diagnostic guidelines [50], there was a lack of diagnostic indicators and training work, suggesting that we must improve the unified criteria. According to the results, the pooled positive rates of clinical signs were observed to provide symptomatic diagnoses and treatment for SFTS patients.

The SFTS patients were classified into mild, severe or mild, common, severe and critical by clinical features and laboratory parameters in previous studies [26, 51]. In our study, routine laboratory parameters of mild and severe cases were analysed. Compared to patients with mild symptoms, the LDH, ALT, AST, and CK levels of patients with severe symptoms were elevated, but albumin and platelet levels were reduced. Leukocytopenia was an important characteristic for SFTS. Data for WBC counts were extracted and analysed, but the scarceness of the literature resulted in no differences between the patients with severe and mild symptoms. Because fever, fatigue, nausea, anorexia, and myalgia were the most frequent symptoms, they could represent the majority of patient conditions. Laboratory parameters and clinical symptoms could serve as references for disease classification and suspected patients.

The risk factors of SFTS patients were evaluated, including the risk factors of poor prognosis and the risk factor of contracting SFTS. Age was an important factor associated with SFTS disease and was a critical risk factor or determinant of morbidity and mortality in SFTS. Farmers accounted for the overwhelming majority of SFTS cases, and farming was a risk factors for SFTS. The transmission of SFTS occurs via tick bites, farmers often work in fields; thus, it is possible that the probability of tick bites was greatly increased for farmers. Further analysis indicated that the occurrence of SFTS was related to tick bites but there was no association between tick bites and fatal outcomes. The reason for the strong heterogeneity might be that the distribution of ticks was different country to country. The six studies originated from 3 countries, including Japan (1 study), Korea (2 studies), and China (3 studies). Tick bites were a risk factor for the occurrence of SFTS, but the deaths of the patients were related to personal conditions and the severity of the disease.

The case-fatality rate of SFTS has varied widely among endemic areas. The case fatality rates in Japan and Korea were apparently higher than that in China [14, 15, 33]. The reasons for this discrepancy might include different notification systems and monitoring durations among the three countries, but the high case fatality from Japan or Korea, the most severe cases reported, just as China at an early stage of SFTS outbreak. We conducted subgroup analysis by region in the three countries. The significant heterogeneity derived from Korea. Because too little literature regarding Korea was included, we could not perform further analysis.

Compared to the previous articles, our meta-analysis had two different levels [52–54]. We summarized the distribution of clinical symptoms, and it was important to distinguish whether the clinical symptoms of each report occurred at the time of hospital visits, at the time of hospitalization or during hospitalization. Fatal and non-fatal patients on clinical symptoms and risk factors, and patients with severe and mild symptoms on routine laboratory parameters, were analysed; thus, we could draw more comprehensive conclusions.

This meta-analysis had some limitations. First, significant heterogeneity brought into question the suitability of performing this meta-analysis, however, the sensitivity analysis showed that the pooled rates were stable and no publication bias was found in our meta-analysis. Second, we could not analyse some indicators in these studies due to lacking data for clinical signs of patients with severe and mild symptoms. Third, the quality of the primary studies might have an impact on the results.

## Conclusions

In conclusion, China was the country with the highest incidence of SFTS. May to July was peak of the epidemic, and farmers were a high-risk group. The risk factor of SFTS included age (poor prognosis) and tick bites (contracting SFTS). Patients with diarrhea and vomiting symptoms on admission should be noted to prevent the disease from aggravating. Clinicians could use the routine laboratory parameters (AST, ALT, LDH, CK, albumin, platelet count) and clinical symptoms (fever, fatigue, nausea, anorexia, myalgia) as references for clinically suspected cases, classification of SFTS and timely treatment, especially in basic hospitals. In addition, epidemiological (population distribution, regional distribution, and time distribution) and clinical characteristics should be combined and then developed public-health interventions for the control and prevention of SFTS.

## Supplementary information

**Additional file 1: Table S1.** The meta-analysis of clinical signs in two groups.

**Additional file 2: Table S2.** The main clinical symptoms of SFTS patients on admission in this review.

## Data Availability

Not applicable.

## Abbreviations

SFTS: Severe fever with thrombocytopenia syndrome
SFTSV: Severe fever with thrombocytopenia syndrome virus
HYSV: Huaiyangshan virus
NBV: New bunyavirus
SMD: Standard mean difference
MD: Mean difference
SD: Standard deviation
OR: Odds ratio
CI: Confidence intervals
LDH: Lactic acid dehydrogenase
ALT: Alanine aminotransferase
AST: Aspirate aminotransferase
CK: Creatine kinase
WBC: White blood cell
