# Supplementary material for: Severe fever with thrombocytopenia syndrome: a systematic review and meta-analysis of epidemiology, clinical signs, routine laboratory diagnosis, risk factors, and outcomes
Source: BMC Infect Dis. 2020 Aug 5;20:575. doi: 10.1186/s12879-020-05303-0 (PMC7409422; doi:10.1186/s12879-020-05303-0)
Supplement: Supplementary file 1 — Additional file 1: Table S1. The meta-analysis of clinical signs in two groups. [file 12879_2020_5303_MOESM1_ESM.docx]

**Table S1** The meta-analysis of clinical signs in two groups

| Symptoms | On admission |  | During the hospitalization |
| --- | --- | --- | --- |
|  | OR (95% CI) P value |  | OR (95% CI) P value |
| Myalgia | 1.05 (0.70-1.58) 0.82 |  | 0.70 (0.37-1.33) 0.28 |
| Anorexia | 1.03 (0.56-1.88) 0.93 |  | 0.84 (0.43-1.66) 0.62 |
| Nausea | 1.42 (0.93-2.17) 0.10 |  | 0.57 (0.30-1.10) 0.09 |
| Abdominal pain | 1.14 (0.67-1.94) 0.62 |  | 1.56 (0.68-3.62) 0.30 |
| Diarrhea | 1.60 (1.06-2.42) 0.02 |  | 1.43 (0.75-2.72) 0.28 |
| Vomiting | 1.56 (1.01-2.39) 0.04 |  | 0.95 (0.48-1.89) 0.88 |
| Sputum | 1.01(0.38-2.69) 0.98 |  | 0.90 (0.38-2.10) 0.80 |
| Headache | 0.83 (0.53-1.31) 0.43 |  | 0.92 (0.48-1.77) 0.81 |
| Fatigue | 1.66 (0.93-2.96) 0.09 |  | 0.91 (0.32-2.55) 0.86 |
| Cough | 1.13 (0.24-5.38) 0.88 |  | 0.58 (0.20-1.65) 0.31 |
| Petechiae | 2.51 (1.00-6.33) 0.05 |  | 3.42 (0.74-15.74) 0.11 |
| Gingival bleeding | 1.11 (0.69-1.80) 0.67 |  | 0.95 (0.39-2.33) 0.92 |
| Lymphadenopathy | 0.88 (0.59-1.30) 0.51 |  | 0.62 (0.25-1.35) 0.31 |
