# Supplementary material for: Severe fever with thrombocytopenia syndrome: a systematic review and meta-analysis of epidemiology, clinical signs, routine laboratory diagnosis, risk factors, and outcomes
Source: BMC Infect Dis. 2020 Aug 5;20:575. doi: 10.1186/s12879-020-05303-0 (PMC7409422; doi:10.1186/s12879-020-05303-0)
Supplement: Supplementary file 2 — Additional file 2: Table S2. The main clinical symptoms of SFTS patients on admission in this review. [file 12879_2020_5303_MOESM2_ESM.docx]

**Table S2** The main clinical symptoms of SFTS patients on admission in this review

| Symptoms | No. Studies. | Meta-analysis, pooled rate (95% CI) | Heterogeneity, P-value (I-squared) | Sensitive analysis (remove single case data set) ^b^ | Heterogeneity, P-value (I-squared) ^b^ |
| --- | --- | --- | --- | --- | --- |
| Fever ^a^ | 14 | 0.76(0.62-0.91) | 0.00(96.8%) | 0.76(0.62-0.91) | 0.00(96.8%) |
| Myalgia | 13 | 0.53(0.41-0.65) | 0.00(98.4%) | 0.50(0.30-0.71) | 0.00(98.6%) |
| Anorexia | 7 | 0.77(0.67-0.88) | 0.00(96.0%) | 0.77(0.63-0.91) | 0.00(95.9%) |
| Nausea | 13 | 0.51(0.41-0.60) | 0.00(95.3%) | 0.48(0.35-0.61) | 0.00(95.0%) |
| Abdominal pain | 10 | 0.25(0.17-0.33) | 0.00(95.9%) | 0.27(0.16-0.39) | 0.00(94.7%) |
| Diarrhea | 14 | 0.45(0.29-0.62) | 0.00(98.8%) | 0.47(0.30-0.63) | 0.00(97.7%) |
| Vomiting | 13 | 0.38(0.26-0.51) | 0.00(97.7%) | 0.39(0.21-0.56) | 0.00(97.9%) |
| Fatigue | 12 | 0.77(0.43-1.12) | 0.00(99.9%) | 0.86(0.81-0.91) | 0.00(87.5%) |
| Sputum | 2 | 0.34(0.19-0.49) | 0.067(70.3%) | 0.24(0.07-0.41) | 0.00(0.00%) |
| Headache | 14 | 0.34(0.23-0.44) | 0.00(96.4%) | 0.35(0.26-0.44) | 0.00(89.4%) |
| Cough | 5 | 0.30(0.12-0.48) | 0.00(96.7%) | 0.24(0.10-0.38) | 0.00(87.1%) |
| Petechiae | 8 | 0.19(0.07-0.31) | 0.00(97.2%) | 0.22(0.09-0.35) | 0.008(94.7%) |
| Gingival bleeding | 7 | 0.09(0.06-0.13) | 0.002(71.8%) | 0.09(0.05-0.14) | 0.004(71.1%) |
| Lymphadenopathy | 11 | 0.42(0.35-0.49) | 0.00(86.3%) | 0.41(0.35-0.47) | 0.00(67.3%) |

^a^Fever means the temperature ≥39℃ in references (37, 40, 41, 43); ≥38℃ in reference (33); the others only mentioned fever. ^b^means remove the forty-fourth reference data set.
